# Graphene-on-gold surface plasmon resonance sensors resilient to high-temperature annealing

**DOI:** 10.1007/s00216-022-04450-4

**Published:** 2022-11-30

**Authors:** Robert Jungnickel, Francesca Mirabella, Jörg Manfred Stockmann, Jörg Radnik, Kannan Balasubramanian

**Affiliations:** 1 Department of Chemistry, School of Analytical Sciences Adlershof (SALSA) & IRIS Adlershof, Humboldt-Universität Zu Berlin, 10117 Berlin, Germany; 2 Federal Institute for Materials Research and Testing (BAM), Richard-Willstätter-Str. 11, 12489 Berlin, Germany; 3 Present Address: SPECS Surface Nano Analysis GmbH, Voltastr. 5, 13355 Berlin, Germany

**Keywords:** Surface plasmon resonance, Graphene, Annealing, Sensing, Surface regeneration, Chromium etching

## Abstract

Gold films coated with a graphene sheet are being widely used as sensors for the detection of label-free binding interactions using surface plasmon resonance (SPR). During the preparation of such sensors, it is often essential to subject the sensor chips to a high-temperature treatment in order to ensure a clean graphene surface. However, sensor chips used currently, which often use chromium as an adhesion promoter, cannot be subjected to temperatures above 250 °C, because under such conditions, chromium is found to reorganize and diffuse to the surface, where it is easily oxidized, impairing the quality of SPR spectra. Here we present an optimized preparation strategy involving a three-cycle tempering coupled with chromium (oxide) etching, which allows the graphene-coated SPR chips to be annealed up to 500 °C with little deterioration of the surface morphology. In addition, the treatment delivers a surface that shows a clear enhancement in spectral response together with a good refractive index sensitivity. We demonstrate the applicability of our sensors by studying the kinetics of avidin–biotin binding at different pH repeatedly on the same chip. The possibility to anneal can be exploited to recover the original surface after sensing trials, which allowed us to reuse the sensor for at least six cycles of biomolecule adsorption.

**Supplementary Information:**

The online version contains supplementary material available at 10.1007/s00216-022-04450-4.

## Introduction

Surface plasmon resonance (SPR) sensors are powerful tools for detecting and determining the specificity, affinity, and kinetic parameters of intermolecular interactions [1–4]. Such sensors typically comprise of a metal film like gold, silver, or copper to excite surface plasmon polaritons, which are very sensitive to changes in the refractive index near the metal surface [5]. In the Kretschmann configuration with a prism-coupled gold surface, where the SPR response is often recorded as angular spectra, it is reported that using gold at a thickness of 50 nm has the best sensitivity [6]. For the use of evaporated gold on glass, usually a thin chromium (or titanium) interlayer as an adhesion promoter is needed to ensure reliable attachment of the gold layer onto the glass substrate. Although the chromium layer is usually much thinner compared to the gold layer (values for SPR-Au chips used in this study are 48 nm Au on top of 2 nm Cr), its influence on the angular SPR spectrum (SPS) is not negligible and results in damping of the SPS curve. This can be attributed to a mismatch in the imaginary part ($\epsilon "$) of the relative permittivity between Au and Cr at ${\lambda }_{\text{ex}}$ = 632.8 nm ($\epsilon "=1.3$ for Au vs. $\epsilon "=20$ for Cr) [7, 8], which is associated with a difference in the absorption of light at this wavelength [9].

Another problem that occurs with this adhesion metal layer is surface reorganization due to diffusion phenomena, when heating the Au/Cr film above certain temperatures [10–13]. In SPR chips, this leads to degradation of the gold surface and alteration of the SPS curve in an unfavorable direction. We show that such problems also apply if a graphene monolayer is transferred on top of the gold surface and the chip is subjected to an annealing step at high temperatures. Because of its unique properties, graphene is widely used as a sensor or electrode material that additionally provides performance enhancement effects when transferred onto SPR-Au sensors [14–17]. Several applications for graphene devices like field effect transistors or electrochemical sensors require a clean graphene surface free of parasitic doping effects [18, 19]. Such effects may be induced by surface adsorbates, e.g., from residues of the polymer used for graphene transfer [20–22]. Furthermore, when working with aromatic molecules, pi-pi stacking phenomena can lead to very tenacious adsorption onto the graphene [23–25] causing a stronger background signal or unwanted electrochemical effects when using the graphene as a working electrode. To recover a clean graphene surface after such adsorption or from parasitic effects, annealing of the graphene is a very efficient strategy [26, 27]. Here we describe a method, which allows for the annealing of transferred graphene on SPR-Au chips while maintaining the SPS quality. With such sensors, we demonstrate a proof-of-principle by following the kinetics of biotin–avidin interaction, wherein the same chip can be reused several times to obtain a nearly identical sensor response.

## Experimental section

The experimental details are provided in Electronic Supplementary Material (ESM).

## Results and discussion

**Fig. 1:**
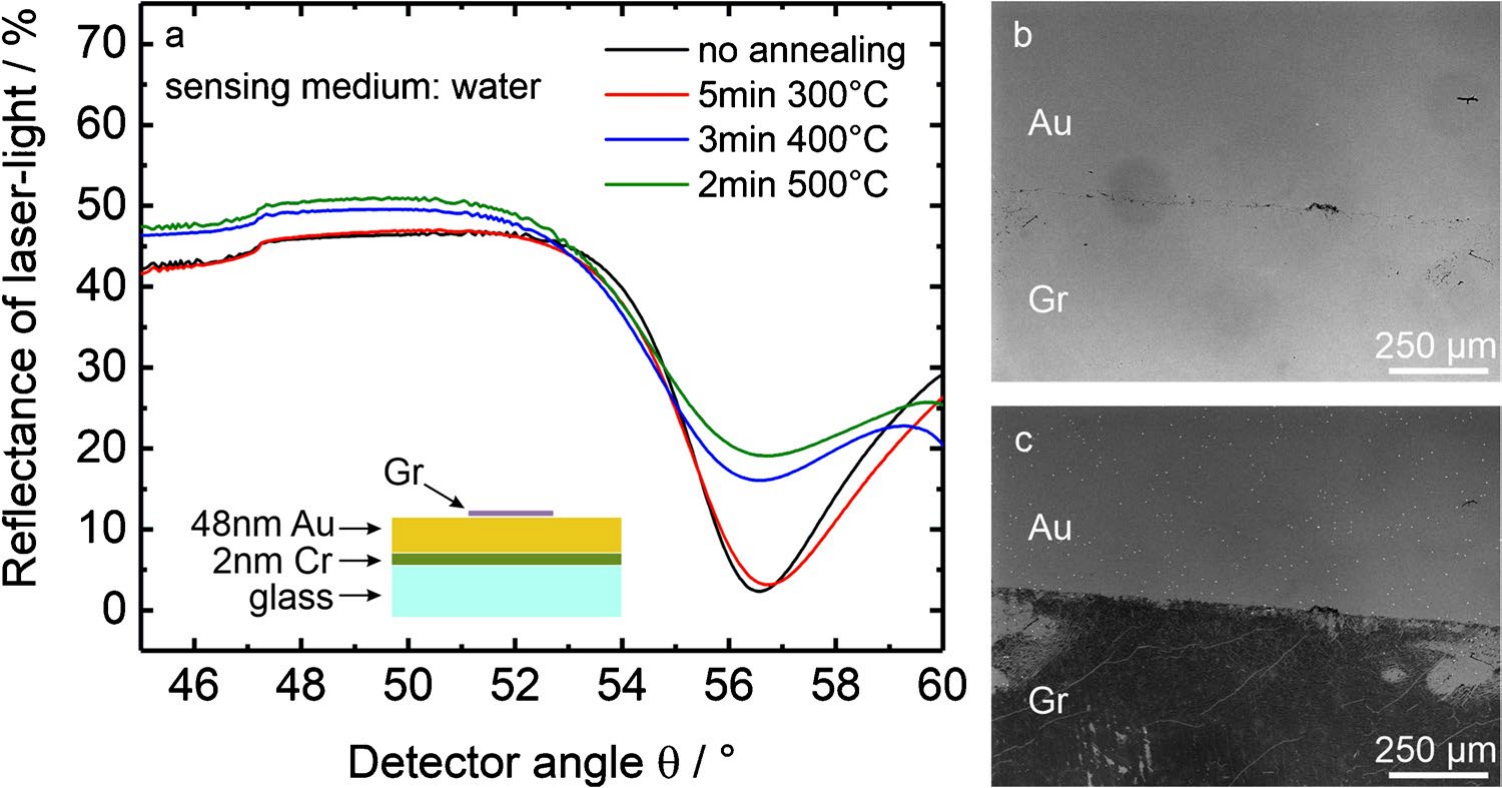
**a** Reflectance spectra at SPR-Au/Gr chips in water without annealing (black curve), and after annealing under nitrogen atmosphere at the indicated temperatures. The scheme in the inset shows the different layers of the SPR-Au/Gr chips. **b**, **c** Optical images (in transmission mode) of an SPR-Au/Gr chip showing the same graphene peripheral region before (**b**) and after (**c**) annealing to 500 °C for 5 min under nitrogen atmosphere. Holes in the gold layer can be identified as white dots in **c**. See figure S1 in ESM for a close-up image

We first present the problem related to the annealing of as-prepared graphene-coated Au SPR (SPR-Au/Gr) sensors (prepared on soda-lime glass) to temperatures above 250 °C in a nitrogen atmosphere. The experimental details can be found in Sect. 1 in Electronic Supplementary Material (ESM). Figure 1a presents angular SPR spectra (measured in water) before and after heat treatment at three different temperatures: 300 °C, 400 °C, and 500 °C. It can be observed that there are only minor changes in the spectrum for an annealing temperature of 300 °C, while for 400 °C and 500 °C, the SPS curve shifts to higher reflectance values, hinting towards an inferior sensor surface. This can be qualitatively inferred from a strong broadening of the SPS-dip and a relative increase in the minimum reflectance (*I*_min_). Optical images of the sample before (Fig. 1b) and after being heated to 500 °C (Fig. 1c) show cracks developing in the graphene sheet as well as a change in optical contrast. Most importantly, the graphene-free gold surface (upper half) appears to be altered, with several holes appearing in the graphene-free gold region (more clearly visible in the close-up images in figure S1 in ESM). Also with titanium as an adhesion layer, such holes are observed on heat-treated SPR chips (see figure S2 in ESM). The time of heat treatment was kept low in these experiments to limit possible stress caused to graphene. Ideally, we would like to have the same surface morphology without changes in SPR spectra after annealing, which is clearly not the case here. Using systematic SPR measurements with associated simulations together with optical and AFM images (as discussed in detail in Sect. 2 in ESM), we have identified the reason for these annealing-induced changes. The observed problems are attributed to heat-induced intermigration of Cr in the Au/Cr layer followed by subsequent oxidation and accumulation of chromium/chromium oxide on the sensor surface. Also in the presence of graphene, the migration of Cr is found to occur; however, the oxidation occurs to a smaller extent due to the protective graphene layer. This intermigration causes a strong deterioration of the SPS spectra when SPR-Au/Gr sensors are subjected to high-temperature annealing.

**Fig. 2:**
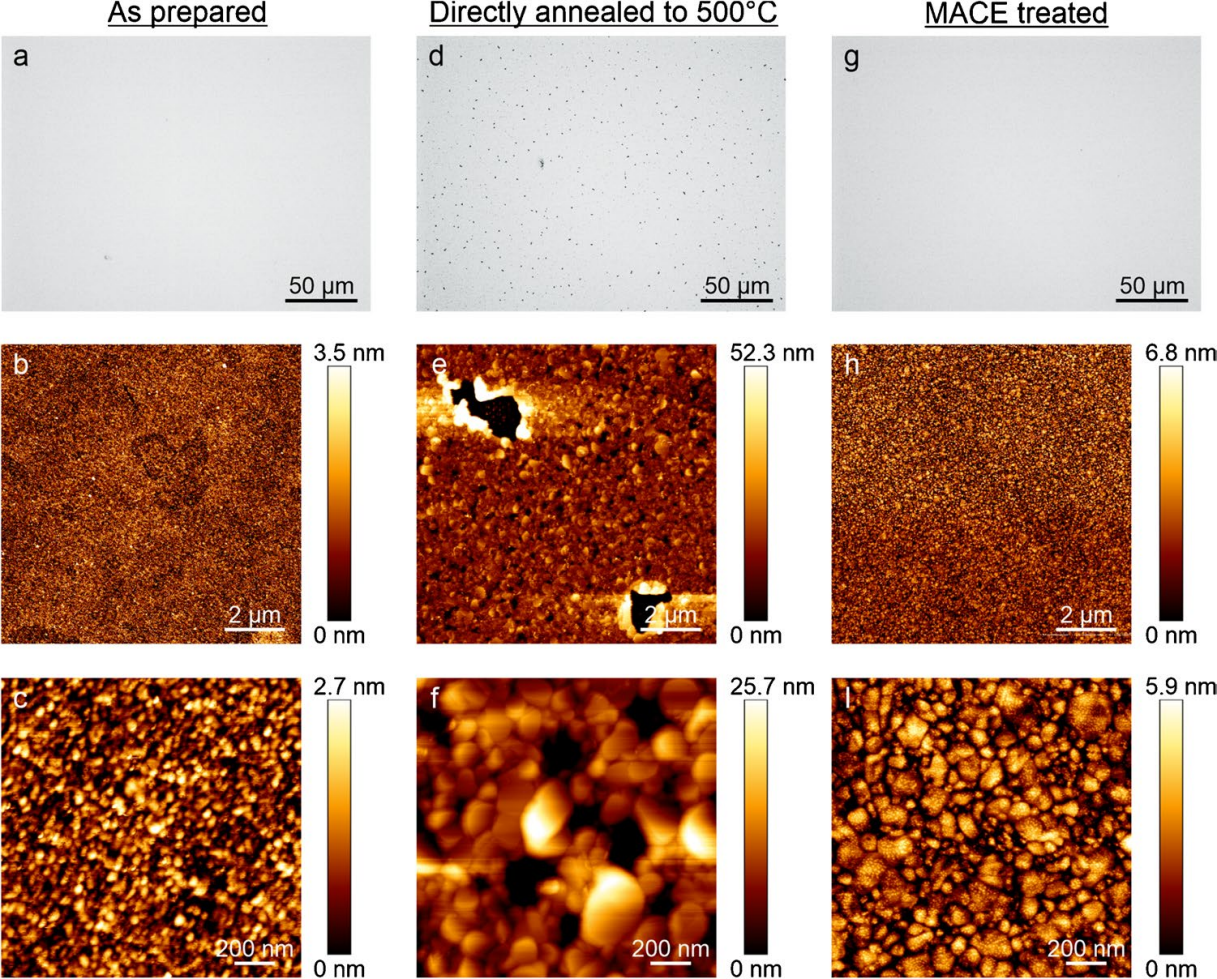
Optical (first row) and AFM (second and third rows) images of the gold surface of SPR-Au chips: **a**–**c** unheated; **d**–**f** directly annealed at 500 °C and Cr-etched; **g**–**i** prepared using the proposed MACE procedure

For overcoming this problem, we need a heat-treatment strategy that avoids hole formation on the gold surface. At the same time, we have to ensure that the chromium/chromium oxide (Cr(Ox)) migrated to the sensor surface is removed before graphene transfer. To obtain a closer look at the morphology of the holes, optical and AFM images were obtained on the same SPR-Au chip (without graphene) before (Fig. 2a–c) and after (Fig. 2d–f) the heat treatment. We infer from the AFM images that SPR-Au chips without any heat treatment typically show gold grain sizes of around 50 nm in diameter (Fig. 2b–c), while the RMS roughness is about 0.6 nm. After the heat treatment and etching, holes are formed and the RMS roughness increased to around 6 nm with the gold grains being much less uniform in size reaching diameters of over 400 nm (Fig. 2e–f). We can also see additional material that is collected around the microholes like a crater (see Sect. 2 in ESM for more details). We found that an important factor to reduce the size and amount of holes is to minimize the leftovers of oxygen traces by thoroughly flooding the heating chamber with nitrogen for several minutes before heating. After several different attempts (see details in Sect. 3 in ESM), successful prevention of hole formation and Cr(Ox) removal could be achieved by a three-cycle heating-and-Cr(Ox)-etching procedure: heating the sample to 300 °C (5 min in N_2_) followed by Cr(Ox)-etching and repeating the procedure at 400 °C and then again at 500 °C. We refer to this optimized annealing treatment as multi-cycle annealing coupled to chromium etching (shortly MACE treatment). The Cr(Ox) etching is carried out in a ceric ammonium nitrate–based etchant solution according to the reaction:

$$3\;\mathrm{Ce}({\mathrm{NH}}_4)_2({\mathrm{NO}}_3)_6+\mathrm{Cr}\rightarrow\mathrm{Cr}({\mathrm{NO}}_3)_3+3\;\mathrm{Ce}({\mathrm{NH}}_4)_2({\mathrm{NO}}_3)_5$$

Using this method, chromium and its oxides can be easily etched away without harming the gold layer. Figure 2g–i shows optical and AFM images obtained on samples subjected to MACE treatment, where it is apparent that there are no holes generated. From the AFM pictures, we infer that the RMS roughness increased only to 1.4 nm, while the gold grain sizes are still heterogeneous reaching diameters up to 200 nm. Nevertheless, graphene transferred on to such chips could be subsequently annealed without any problem of surface reconstruction or morphology changes (see figure S5 in ESM), yielding an SPR spectrum of very good quality, as will be discussed later. The adhesion of the gold layer onto the glass of the SPR-Au chips that were annealed following the MACE protocol was tested by pressing a Scotch tape onto the chip and removing it after 30 s [28]. The removed Scotch tape showed no leftovers of gold even after storage of the MACE-treated SPR-Au chips for a month ensuring sufficient stability of the sensor. The etching of surface-migrated chromium (oxide) could also be achieved electrochemically (see figure S6 in ESM).

The exact reasons behind the drastically low density of microholes when using the MACE treatment (in comparison to the appearance using a direct annealing at 500 °C) are not completely clear. We have observed that SPR-Au chips, whose gold surface had a higher surface roughness (RMS roughness of about 4 nm before any heat treatment) to begin with, showed significantly less holes after heating them directly to 500 °C (figure S7 in ESM). Such rough surfaces are not well-suited for graphene transfer since a proper surface characterization using e.g. AFM is not possible due to the high roughness. Diffusional transport in solid metals can appear by various mechanisms always involving some kind of defects such as vacancies, dislocations, and grain boundaries [29, 30]. For thin film interdiffusion, a distinction between three kinetic regimes can be made: pure grain boundary diffusion, atomic migration from the boundaries into the grains, and limited diffusion from the grain boundaries into the grains [31, 32]. As the occurrence of a specific diffusion mechanism is typically temperature dependent and intermetallic compound phases may arise at the diffusion zone, it is difficult to find a simple explanation for the aforementioned findings [29, 33]. The absence of microholes in samples of higher initial roughness after heating to 500 °C could be explained via a higher porosity of the gold layer allowing for a more regular migration along the grain boundaries avoiding accumulation and/or stress formation below the gold surface. Similarly, it can be expected that the MACE approach takes away diffused chromium in smaller proportions sequentially, thereby circumventing local accumulation of chromium in large quantities avoiding excessive stress creation, thereby minimizing hole formation. Performing all the three cycles at a fixed temperature of 300 °C did not show the desired result, justifying the requirement of successively higher temperatures in our MACE approach. Furthermore, the MACE treatment also yielded stable Au/Cr layers without holes, when using BK7 glass as the underlying substrate. Clear evidence for the migration and oxidation of chromium in the Au/Cr films and the removal by our etching method is obtained by X-ray photoelectron spectroscopy (XPS) and time-of-flight secondary ion mass spectrometry (TOF–SIMS) measurements as discussed in detail in Sect. 4 in ESM.

**Fig. 3:**
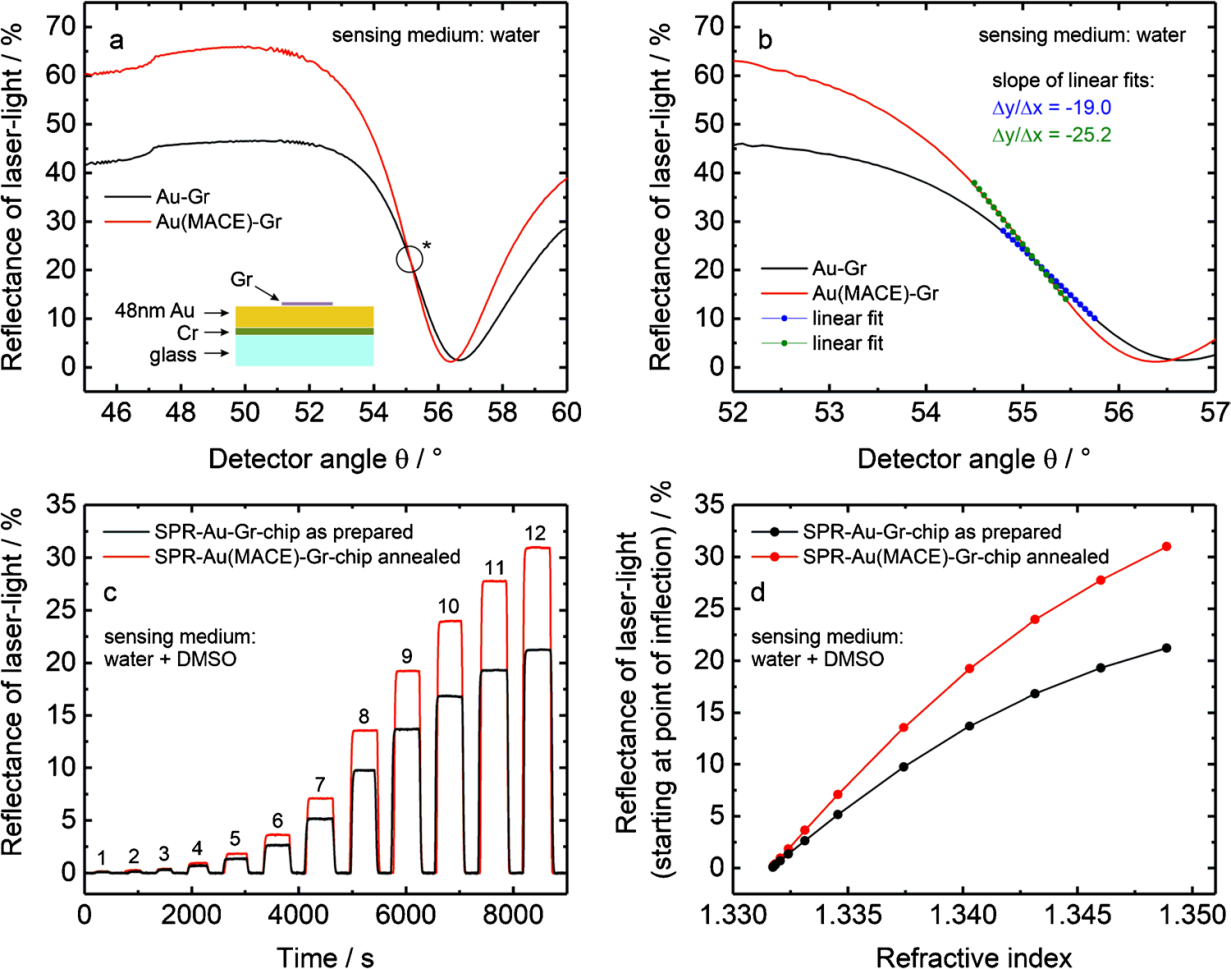
**a**, **b** Reflectance spectra in water for an SPR-Au/Gr chip that is as-prepared unheated (black curve) and after MACE treatment and annealed (red curve). **b** A section of the curves in **a** with linear fit around the point of inflection for both curves to determine the slope. **c** SPR sensorgrams measured at an SPR-Au/Gr chip (with and without MACE treatment) in water at the point of inflection of the SPR spectrum (as indicated with * in **a** for consecutive injections of water-DMSO mixtures with increasing refractive index) (see Table S3 in ESM for details). **d** Calibration curve showing the shift in reflectance (from **c**) as a function of refractive index

Now we turn towards the effect of MACE treatment on the SPR response to obtain an idea of the resulting sensitivity. Figure 3a and b compare the SPR spectrum obtained on an as-prepared SPR-Au/Gr chip with the spectrum obtained after the MACE treatment. The MACE-treated Au/Gr chip was also annealed (500 °C for 2 min under N_2_) after the transfer. The spectrum for this case (red curve in Fig. 3a) demonstrates clearly that a larger reflectance range is accessible with the graphene-coated MACE-treated chip without any deterioration of dip width or *I*_min_. This suggests that the issue of degradation of the SPS curve after annealing is now completely prevented (compare with Fig. 1a). Most importantly, the angular sensitivity is clearly improved by the MACE treatment, as shown in Fig. 3b. The sensitivity towards refractive index was further evaluated by recording SPS curves and sensorgrams (reflectance at a constant angle) in DMSO-water solutions in varying proportions. Figure 3c compares sensorgrams measured on untreated SPR-Au/Gr chips to those that were subjected to MACE treatment and subsequently annealed. The sensorgrams show the temporal changes in reflectance upon consecutive injections of increasing proportion of DMSO corresponding to a stepwise increase in refractive index (see Table S3 in ESM for the exact values of refractive index). Calibration curves extracted from these datasets are shown in Fig. 3d, where it is apparent that the annealed SPR-Au chips showed a better sensitivity than the untreated chips. It is worth mentioning that the sensitivity of the resonance angle to refractive index changes (*S*) was maintained also in the MACE-treated SPR-Au/Gr chips (see figure S10 in ESM). A representative summary of the different quality parameters underlining the suitability of the proposed MACE treatment is presented in Table S4 in ESM.

**Fig. 4:**
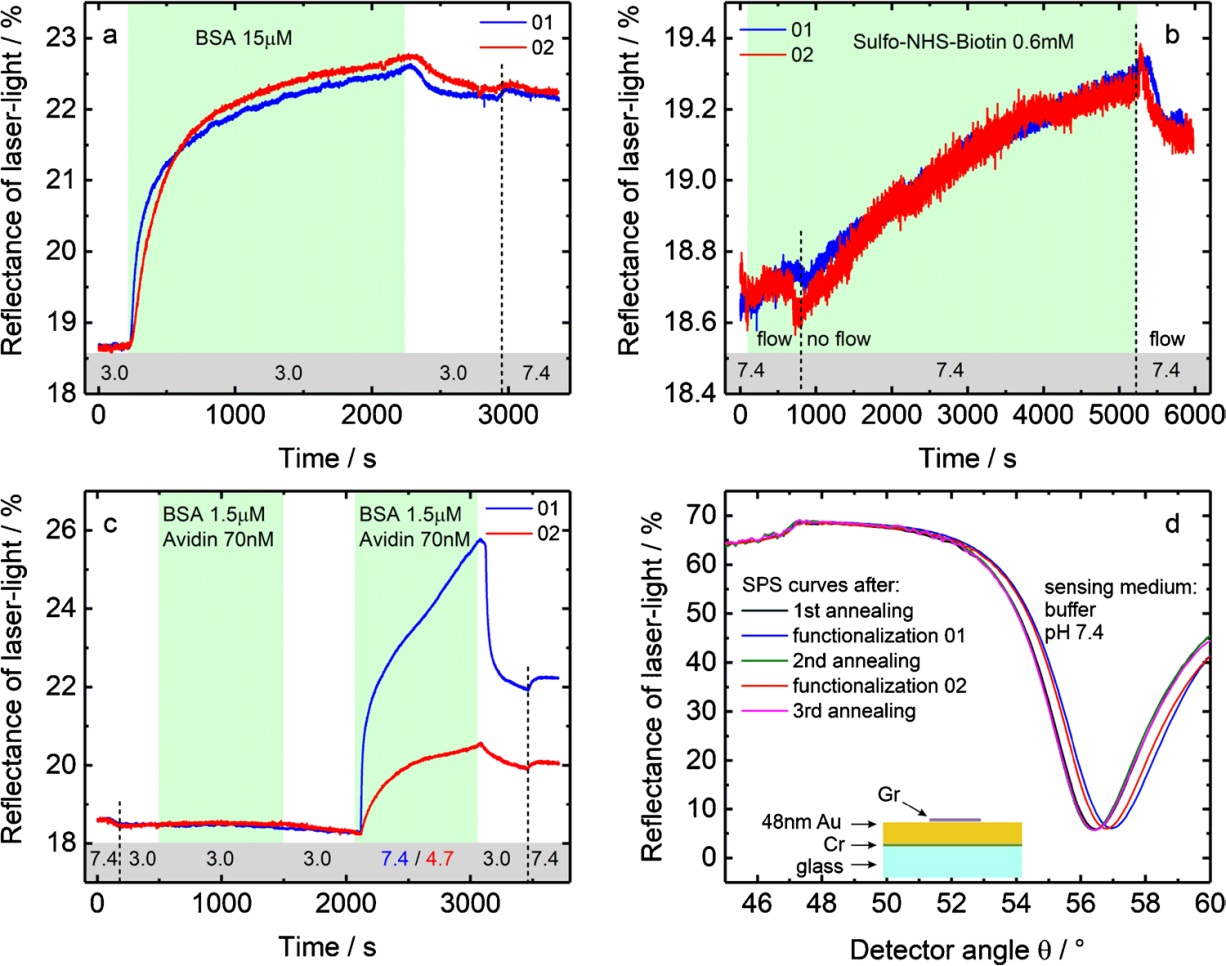
**a**–**c** SPR sensorgrams measured at an SPR-Au/Gr chip prepared using the MACE procedure: **a** attachment of BSA to graphene. **b** Coupling of sulfo-NHS-biotin to the BSA-graphene surface. **c** Interaction of avidin with the biotin-BSA-graphene surface. The blue curves show the results from the first measurement cycle, while the red curves show the data obtained on the same chip after annealing the chip to 500 °C for 5 min in N_2_. The pH values of the buffers used are denoted in gray in the bottom of the graphs. **d** Angular reflectance spectra in 10 mM phosphate buffer (pH 7.4) of the same SPR chip at various stages of the sensor trials. After the annealing steps, the original spectrum is completely restored

Finally, we demonstrate an application example using our MACE-treated SPR-Au/Gr sensors by repeated evaluation of a classical biomolecular interaction, namely the binding of avidin to surface-bound biotin on the same sensor chip. Since this binding is one of the strongest with an exceptionally high affinity [34], it serves as a good example to demonstrate the advantages of our MACE approach. The general idea is to couple amine-reactive biotin to a BSA-modified graphene surface and subsequently study the interaction of avidin with this surface at different pH. Two cycles of surface functionalization and binding interaction were carried out on the same sensor chip. After the first cycle, the chip was annealed in order to recover the sensor surface and to evaluate the reusability of the SPR-Au/Gr sensors prepared using our methodology. Figure 4a shows the SPR response during the functionalization of graphene surface using BSA, while Fig. 4b presents the evolution of the response during the biotin-coupling step. The kinetic evolution of the responses during the two cycles for each of the two cases are very similar attesting the possibility to reuse the same sensor chip after high-temperature annealing. The BSA binding to graphene is carried out at low pH, where BSA is positively charged, while graphene is nearly neutral [35]. Hence the attachment of BSA to graphene is mostly through hydrophobic interactions as confirmed by the limited change in SPR response even when moving to higher pH of 7.4 during the final dissociation phase in Fig. 4a. A successful coupling of biotin is inferred by an increase in the SPR signal during the interaction of sulfo-NHS-biotin with the BSA-modified graphene surface (Fig. 4b).

Figure 4c presents the interaction of avidin with the biotin-BSA-modified graphene surface during the two cycles. In both the cycles, during the first half, the interaction is followed at a low pH of 3.0 as a control step. The addition of avidin does not affect the sensor response here, signifying an absence of interaction between avidin and the biotin-BSA-modified graphene surface. This can be understood by considering that at this pH, both avidin (pI ~ 10) and the sensor surface (pIs: biotin — 4.5, BSA — 4.7) are positively charged, leading to electrostatic repulsion. In the second half, the interaction is studied at two different pH (blue curve — pH 7.4 and red curve — pH 4.7). At pH 4.7 (red curve), the modified graphene surface is expected to be neutral and hence we expect to see mainly the specific interaction between avidin and biotin, as inferred from the classical association curve. By contrast, at pH 7.4 (blue curve), there is a sudden increase in the SPR signal upon addition of avidin followed by a slower increase. The sudden increase can be explained by considering that the avidin molecules and the modified graphene surface are oppositely charged leading to a strong electrostatic attraction. The observed response is thus a superposition of an electrostatic component and a specific interaction component between the analyte and receptor molecules. This is also apparent in the dissociation phase, which was carried out at pH 3.0, where the non-specifically electrostatically bound avidin molecules are washed away very quickly as exemplified by the strong decrease in SPR signal in this phase. We have thus shown that using our carefully prepared graphene surface, which is reusable, we can disentangle non-specific interactions occurring via electrostatic forces from a specific interaction involving the molecular recognition of biotin by avidin.

Finally, Fig. 4d presents the SPS curves at the various steps of this sensor trial. It can be seen that the sensor response shifts to higher angles upon functionalization/surface modification, while after annealing, we are always able to recover the original SPS response with the same quality as we had started out. This confirms that all strongly bound protein species, including the avidin molecules, can be successfully removed without any deterioration in quality using our heat treatment methodology. In SPR-based assays, harsh acid and base treatments or chaotropic agents are often utilized to induce the dissociation of irreversibly bound analyte species [1]. While an acid treatment is still suitable for our graphene samples, other treatments cannot be utilized, because they lead often to graphene rollup and the creation of cracks and holes in the one-atom-thick carbon sheet. Although the MACE treatment requires removing the SPR chip from the flow cell, the ability to recover the original surface is highly advantageous for repetitive use of the same SPR chip for several sensor trials. The need for repetitive use is also crucial to obtain reliable calibration curves by measuring the sensor response with suitable calibration standards on the same sensor chip with the same initial surface conditions. We were successful in recovering the same angular SPR response even after 10 cycles of annealing (see figure S11 in ESM). We also performed biodetection in a diluted serum sample spiked with avidin (see figure S12 in ESM), after which we could regenerate the surface to a good extent by using a slightly increased annealing time of 10 min. When performing biodetection on the graphene surface in standard solutions, the regeneration of the surface and the recovery of the kinetic response were possible even after 6 cycles (see figure S13 in ESM) with an annealing time of 5 min.

## Conclusion

In summary, we have identified the problems occurring on SPR-Au and SPR-Au/Gr sensors, when subjected to high-temperature annealing. We observed that (inter)diffusion of the metallic layers leads to creation of holes and deterioration of the SPS characteristics. In order to overcome this problem, MACE has been introduced as an optimized preparation strategy, which involves multiple (3) cycles of annealing followed by chromium (oxide) etching. By using this procedure, we have shown that the morphology of the Au surface remains rather unaffected, delivering an enhanced SPR performance as characterized by a clear improvement in angular sensitivity, larger dip strength, and superior refractive index sensitivity. Moreover, we showed that the high quality of the SPR response is preserved despite adding an optically absorptive layer like graphene on the gold surface. Most importantly, this opens up the pathway to anneal graphene-coated SPR chips, which can ensure the availability of a clean *sp*^2^-carbon surface for studying biomolecular interactions. The capability to anneal the samples also allowed for a complete recovery of the sensor surface even after the strong binding of avidin to a biotin-functionalized graphene surface. This feature will allow for a high degree of reusability of the graphene SPR sensor chips.

## Supplementary Information

Below is the link to the electronic supplementary material.

Supplementary file1 (PDF 2921 KB)
