# Supplementary material for: Graphene-on-gold surface plasmon resonance sensors resilient to high-temperature annealing
Source: Anal Bioanal Chem. 2022 Nov 30;415(3):371–7. doi: 10.1007/s00216-022-04450-4 (PMC9829571; doi:10.1007/s00216-022-04450-4)
Supplement: Supplementary file 1 — Supplementary file1 (PDF 2921 KB) [file 216_2022_4450_MOESM1_ESM.pdf]

Graphene-on-gold surface plasmon resonance sensors resilient to high-temperature annealing

Robert Jungnickel [1,], Francesca Mirabella [2§], Jörg Manfred Stockmann [2], Jörg Radnik [2],

Kannan Balasubramanian [1*],

[1] Department of Chemistry, School of Analytical Sciences Adlershof (SALSA) & IRIS Adlershof, Humboldt-Universität zu Berlin, 10117 Berlin, Germany.

[2] Federal Institute for Materials Research and Testing (BAM), Richard-Willstätter-Str. 11, 12489 Berlin, Germany.

[§] Current Address: SPECS Surface Nano Analysis GmbH, Voltastr. 5, 13355 Berlin.

Corresponding author e-mail: nano.anchem@hu-berlin.de

ELECTRONIC SUPPLEMENTARY MATERIAL

1. Experimental Section
2. Effect of annealing the SPR-Au sensors
3. Development of optimized preparation procedure (MACE)
4. Spectroscopic evidence for the occurrence and removal of surface chromium (oxide) species

1. Experimental Section

Chemicals. Potassium chloride (≥ 99.0 %, Emprove® Essential), sulfuric acid (98 w%, p.a., Emsure®), hydrochloric acid (37 w%, p.a., Emsure®), polystyrene (PS, av. Mw 35000), chloroform (≥99.8 %, ACS spectrophotometric grade), methanol (for LC-MS LiChrosolv®), toluene (≥ 99.8 %, Rotisolv®) and ceric ammonium nitrate-based chromium etchant standard (MDL: MFCD07370678) were purchased from Sigma-Aldrich. Hydrogen peroxide (30 %, p.a., AnalaR NORMAPUR®), ammonium hydroxide (25 % p.a.), and ethanol (≥99,8 %, AnalaR NORMAPUR®) were from VWR. Meldola's blue was obtained from chempur (MDL: MFCD00012113). Soda-lime glass slides were purchased from Carl Roth. Chemical vapor deposition (CVD-)grown graphene on polished copper foil was purchased from Graphenea. All solutions were prepared with ultra-pure water (Barnstead Easypure II system, 18.2 $M\Omega cm^{-1}$).

Fabrication of SPR-Au chips.
Soda-lime glass microscope slides (76 mm x 26 mm x 1 mm) were used as substrate for the gold-layer. Rigorous cleaning of the glass slides prior to the deposition of the metal layers proved very important to obtain gold layers with a low surface roughness. During each cleaning step, the glass slides were kept apart by a polypropylene microscope slide-holder. The first

cleaning step involved ultrasonication in a hydrochloric acid (37 %)-ethanol (99 %) 1:1 solution for 30 minutes followed by ultrasonication in water for 5 minutes and finally ultrasonication in ethanol (99 %) for another 5 minutes. After blowing each glass slide dry with pressured air, they were visually inspected for flaws like little scratches or persistent contaminations, which lead to the exclusion of the affected glass slides. Afterwards a standard RCA-cleaning of the slides was done: 1. Removal of organic contaminations by ultrasonication for 10 minutes in a freshly prepared solution of water, hydrogen peroxide (30 %) and ammonium hydroxide (25 %) (50:4:1) that was heated to 80 °C. 2. Removal of metal contamination by ultrasonication for 10 minutes in a freshly prepared solution of water, hydrogen peroxide (30 %) and hydrochloric acid (37 %) (50:4:2) that was heated to 80 °C. Finally sonication in water for 10 minutes was done. During each sonication step, the slides were shaken several times so that no bubbles could permanently cover areas on the glass slides. After each sonication step, the supernatant was slowly decanted before pulling out the glass slides under pouring water to avoid recontamination with the removed contaminants. The final step was a short sonication for 5 minutes in spectroscopic grade methanol and blow-dry of each slide with nitrogen gas before storing them in cleaned airtight plastic boxes for microscope slides. The metal layers were deposited by thermal evaporation. The slides were first cleaned in argon plasma in the vacuum chamber, before evaporation of a 2 nm chromium film and a 48 nm gold film. For both deposition steps, a thermal evaporation vacuum station working at a residual pressure of 2×10^{-6} Pa was used. The titanium containing chips were purchased from XanTec bioanalytics GmbH and were composed of borosilicate glass BF33 with an evaporated gold layer of 44 nm thickness on top of an titanium adhesion layer of approximately 1 nm thickness.

Graphene transfer – SPR-Au/Gr chips.

CVD-graphene (Graphenea) was cut into square pieces (typically 1 cm × 1 cm) and a solution of poly(styrene) (PS) (50 mg/mL in toluene) was spotted over the copper foil (PS/graphene/copper/graphene) and dried at 75 °C for 15 minutes. After the deposition of PS, the etching of copper was carried out in a mixture of water, hydrochloric acid (37%) and hydrogen peroxide (30%) (60:6:1 v/v). After transferring the polystyrene-graphene piece into three consecutive beakers of water, it was finally transferred onto the SPR-Au-chips. The SPR-Au-chips were cleaned prior to the transfer in an argon plasma for 2 minutes to remove hydrophobic surface contaminants [1] and thus ensure a successful transfer. After the transfer, the gold-graphene samples were baked in the oven at 75 °C for 15 minutes. The polystyrene layer is removed by toluene treatment for 5 minutes. The SPR-Au/Gr chip is then rinsed with ethanol and blown dry with light air flow. Afterwards, the chips are heated to 150 °C for 5 minutes to ensure complete removal of water or other solvents. Such samples are referred to as as-prepared SPR-Au/Gr sensors. After mounting the sample in the SPR system, the samples were tested to be free of redox active contaminations by cycling the potential for 10 times at 100 mV/s in the range of -0.2V to 0.7V in 100 mM $HClO_4$, before starting the sensor trials.

Annealing-coupled chromium etching procedure

The as-prepared SPR-Au-chips were cleaned with high purity chloroform and ethanol and dried with pressured air. They are then placed in a glass chamber that is flooded with nitrogen gas

constantly and heated to a preset temperature between 200 °C and 500 °C in a muffle furnace for 5 minutes unless stated otherwise. After cooling down (still in nitrogen atmosphere), the SPR-Au-chips are placed in the chromium etching solution for 30 seconds. The Cr-etch-solution is rinsed away from the chip with copious amounts of water and is washed with methanol and dried with pressured air if used for another annealing step.

Surface characterization.
A NanoScope Dimension 3100 atomic force microscope (AFM) in tapping mode or Bruker JPK Nanowizard 4 was used for surface imaging. The AFM images were processed using Bruker JPK Data Processing software and Gwyddion. The roughness (RMS) is evaluated from 1.5 μm x 1.5 μm AFM topography images.

X-ray photoelectron spectroscopy (XPS)
XPS measurements were performed using a Kratos AXIS Ultra DLD with monochromatic Al-Kα radiation with an energy of 1486.6 eV. The samples were placed on a stainless-steel sample holder and fixed with double adhesive carbon tape. The angle between the detector and the sample surface was 90 degrees (normal emission), and the angle between the X-ray source and the sample was 60 degrees. Survey spectra were measured with a pass energy of 80 eV, high resolution spectra with a pass energy of 20 eV. All spectra were processed using the program Unifit (version 2022). A sum of Lorentzian and Gaussian (L–G mixing of 0.3) was used as the fit function in combination with a modified Tougaard background. Quantification was performed normalizing the peak area after subtracting the modified Tougaard background with the Scofield factors for the photoionization cross sections, inelastic mean free pathways and the spectrometer-specific transmission function.

Time-of-Flight Secondary Ion Mass Spectrometry (ToF-SIMS)
ToF-SIMS measurements were carried out by using a TOF-SIMS IV instrument from IONTOF GmbH, Münster, Germany. Investigations were performed in the spectrometry mode using a 25 keV Bi^+ as primary source. The analysis was performed in both negative and positive polarities. The region of interest (ROI) of 100 μm × 100 μm area was scanned in sawtooth mode with 128x128 pixels.

SPR instrumentation
SPR measurements were performed using a RT2005 spectrometer from RES-TEC GmbH. The instrument operates in Kretschmann configuration using a HeNe laser (λ_{ex} = 632.8 nm) in angle scanning mode. The liquid is delivered on to the SPR-sensor surface using a flow channel controlled by a Fluigent 8-channel Flow Control System (MFCS). For recording of the sensograms, a pressure of 70 mbar was applied, which corresponds to a flow rate of 800±10 μL/min. The angular SPR spectra were recorded without any flow after reaching a steady state in the sensogram. The relative reflectance (in %) is calculated as the proportion of the intensity of the light measured in Kretschmann configuration to the total intensity of incident laser light.

Electrochemical setup.

The electrochemical measurements with an SPR-sensor as the working electrode was performed in a standard three-electrode set-up using an Ivium Compactstat potentiostat. A Pt wire was used as the counter electrode, an Ag/AgCl (3 M KCl) leak-less miniature electrode (eDAQ model ET072) served as the reference electrode. Electrochemical SPR (ecSPR) measurements were done by combining this setup with the SPR system described above.

SPR performance parameters and reflectance simulations.

Reflectivity curves as a function of angle were simulated using the program Winspall 3.01 (freely available under http://www.res-tec.de/downloads.html). The software simulates angular SPR curves of a multilayer system based on the Fresnel equations and the transfer matrix method. [2] The refractive index and extinction coefficient (at 633 nm) of the layers used for the simulations are: Cr [3] n = 3.13 k = 3.32; Au [4] n = 0.19 k = 3.5; chromium oxide (CrOx) [5, 6] n = 2.1 k = 0.4. We extracted parameters such as half width half maximum (HWHM), relative intensity at resonance ($I_{\min}$) and angle at resonance (θ_{res}) from the recorded reflectivity curves using Origin. Instead of the Full-Width Half Maximum (FWHM) only the HWHM from the left side (lower angles) of the SPR-curve is used for the performance comparison due to the asymmetry of the angular SPR spectrum. [7] The performance parameters used in this study are the sensitivity (*S*), the detection accuracy (*DA*), the quality factor (*QF*) and the dip strength (*DS*) defined by the following equations:

$$S = \frac{\Delta\theta_{\text{res}}}{\Delta n_{\text{sens}}} \qquad (1)$$

$$QF = \frac{S}{HWHM} \qquad (2)$$

$$DA = \frac{1}{HWHM} \qquad (3)$$

$$DS = I_{\max} - I_{\min} \qquad (4)$$

The sensitivity *S* (unit: deg/RIU, RIU: Refractive Index Unit) is defined as the ratio of the shift in the resonance angle ($\Delta\theta_{\text{res}}$) per unit change in refractive index of the sensing medium (Δn_{sens}). The quality factor (*QF*) (sometimes also referred as figure of merit FOM) relates the sensitivity *S* with HWHM (or FWHM) and is often used to compare different SPR-sensors.[8, 9] The detection accuracy gives a description of how accurately the minimum angle can be determined. Higher values of these parameters are associated with a better performance of the SPR sensor.

Biotin-avidin sensor trials

First, in order to provide primary amino groups, the graphene surface was covered with BSA via unspecific binding. The BSA was adsorbed onto the graphene surface from a 15 µM solution in phosphate-buffer (pH 3.0). For subsequent biotynilation, the surface amino groups of the adsorbed BSA were treated with a 0.6 mM sulfo-NHS-biotin solution in phosphate-buffer (pH 7.4; NHS: *N*-hydroxysuccinimide). After inflow of the solution, the flow was stopped and the reaction was let to take place during the next 5400 sec after which unbound biotin was washed away via inflow of phosphate-buffer (pH 7.4). For studying the binding of the avidin, first a solution of 1.5 µM BSA in phosphate-buffer (pH 3.0) was injected to ensure the coverage of

leftover unspecific binding sites. This was followed by inflow of 70 nM avidin with 1.5 µM BSA in phosphate-buffer (pH 3.0). The solutions contained BSA all along in order to limit non-specific binding. Annealing was carried out at 500°C for 5 minutes in nitrogen atmosphere after each of the two sensing cycles.

Figure S1. A close-up view of the images of SPR-Au/Gr sensors in figures 1b and 1c. Optical images (in transmission mode) of an SPR-Au/Gr chip showing the same graphene peripheral region before (**a**) and after (**b**) annealing to 500 °C for 5 minutes under nitrogen atmosphere. Holes in the gold layer can be identified as white dots in (**b**).

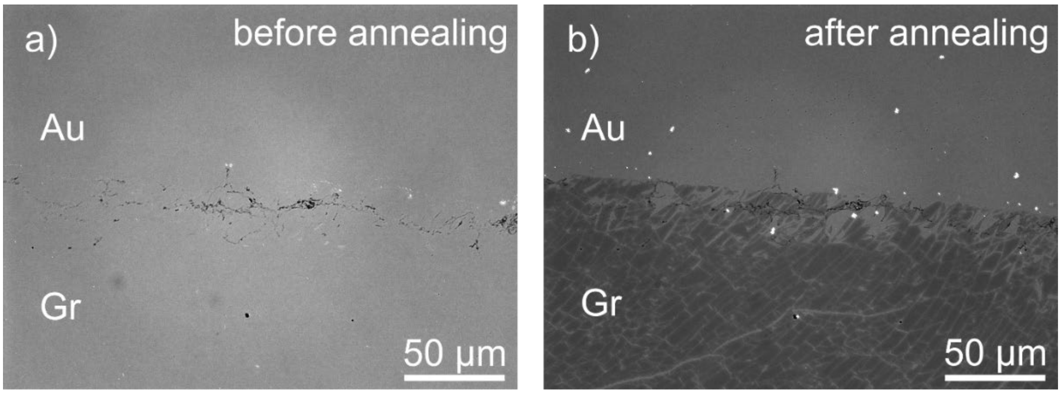

Figure S2. As an alternative for chromium, titanium is often used for an adhesion layer between glass and gold. Upon annealing an SPR-Au chip with Ti as adhesion layer (to 500 °C), holes are formed here too, indicating that we have a similar problem for this case also. The figure below shows optical images of an SPR-Au sensor with Ti as adhesion layer before and after heat treatment (annealing to 500 °C)

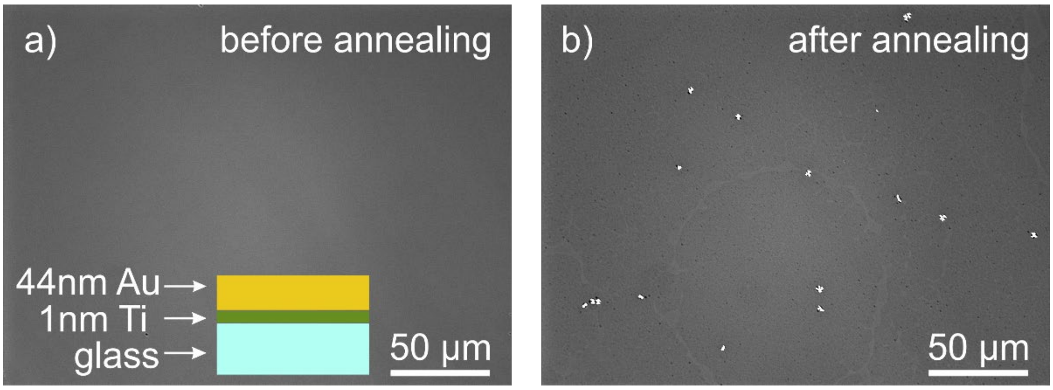

2. Effect of annealing the SPR-Au-sensors

From figure 1 it is clear that the annealing procedure affects the gold-chrromium layer on the glass chip. It is known that graphene itself can withstand higher temperatures without damage.[10, 11] In order to identify the reason for the formation of holes and for the deterioration of the SPR spectra, SPR-Au-chips without graphene were subjected to the same treatment. Figure S3a presents measured SPR spectra on four different chips after heat treatment at different temperatures. While the changes in the spectra are only subtle for temperatures of 300 °C and below, stronger differences are visible in the spectra of chips annealed to higher temperatures. Unlike in figure 1, it can be seen here that there is a clear shift in the resonance angle θ_{res}, while the HWHM increases only slightly, in addition to an increase in I_{max} and I_{min}. SPR-Au chips annealed to 500 °C also contain holes in a rather homogeneous distribution as shown in figure S3b (white spots). In order to understand these observations, it is necessary to take a closer look at the effect of heat treatment of metallic thin films. It is known that at temperatures above 250 °C, chromium starts to diffuse along the grain boundaries of a thin gold layer reaching the surface within a couple of minutes.[12, 13] Moreover, a chromium surface is slowly oxidized in air even under ambient conditions.[14, 15] Indeed, annealed SPR-Au chips, which were left to age for a month, show a halo effect around the holes in the gold layer, as can be seen in the AFM and optical images in figure S3c and S3d respectively. The AFM image shows microholes with a depth of over 40 nm (see inset figure S3c) indicating that they reach down to the surface of the glass slide. The protrusions in the AFM image (figure S3c) around the hole are reminiscent of volcanic eruptions around a crater. Consistent with the knowledge on Au/Cr thin films [12-17], we attribute the formation of holes to an inhomogeneous migration of Cr to the top surface leaving holes behind. In this process, some gold may also be displaced to the upper surface. The halo effect in the optical image (figure S3d) is consistent with a difference in optical transmission arising due to the oxidation of Cr to CrOx around the hole. This halo can also be seen in the AFM image (figure S3c) as a slight elevation of the surface within an radius around the hole of approximately 1 µm. It should also be noted that, despite constant purging of the heating chamber with nitrogen, trace amounts of oxygen cannot be excluded, which may also favor the oxidation of the migrated chromium surface layer in the absence of graphene.

In order to gather support for the proposed migration and oxidation in the Au/Cr layers, we performed a simulation of the SPR spectra with a multilayer geometry using the transfer matrix method. In this procedure, the different metallic layers on the SPR glass chip are modelled with their complex dielectric parameters [5, 6] and layer thickness to simulate the SPR spectrum. Figure S4a presents the SPR spectra for an Au/Cr stack, where the thickness of the chromium adhesion layer is varied from 2 nm (deposited in our as-prepared samples) to 0. It is apparent that as the chromium thickness reduces, mainly I_{max} increases, signifying an improvement in SPR dip strength. In addition, there is a slight decrease in HWHM. As mentioned earlier, chromium just serves as an adhesion layer and as the thickness is reduced, the damping is minimized yielding an improved SPR spectrum. Based on this, we conclude that in both the SPR-Au and SPR-Au/Gr chips, the increase in I_{max} can be correlated with a decrease in chromium amount in the adhesion layer, induced by annealing to high temperatures. In figure

S4b, the effect of migration of chromium to the top of gold is simulated by adding a chromium layer on top of the Au/Cr stack. In order to evaluate the sole effect due to the top chromium layer, we keep the thickness of the adhesion layer constant (0.5 nm). It is clear that as the thickness of the top Cr layer increases, I_{min} shifts to higher reflectance values with a striking increase in HWHM. This is akin to what we observe on the graphene-coated SPR chips (figure 1a). On the other hand, when a CrOx layer is used as a top layer (figure S4c), a clear shift in the resonance minimum (θ_{res}), along with an increase in HWHM is apparent, which is similar to what we observe on the gold SPR chips without graphene (figure S3a). These simulations support well the possibility of migration of chromium from below the gold layer to the top. We expect that the migrated chromium oxidizes when there is no graphene present on top. In the presence of graphene, the oxidation of chromium is hindered and we are left with a rather metallic chromium layer below graphene, explaining the contrast difference in figure 1c.

Figure S3. (**a**) Reflectance spectra of SPR-Au-chips in water without annealing (black curve), and after annealing for 5 minutes under nitrogen atmosphere at the indicated temperatures. (**b**) Optical image of the SPR-Au-chip immediately after undergoing annealing at 500 °C followed by treatment with a Cr-etch solution. (corresponding to the magenta curve in (**a**)). (**c**) AFM and (**d**) optical images of the same SPR-Au-chip obtained one month after the annealing/etching treatment.

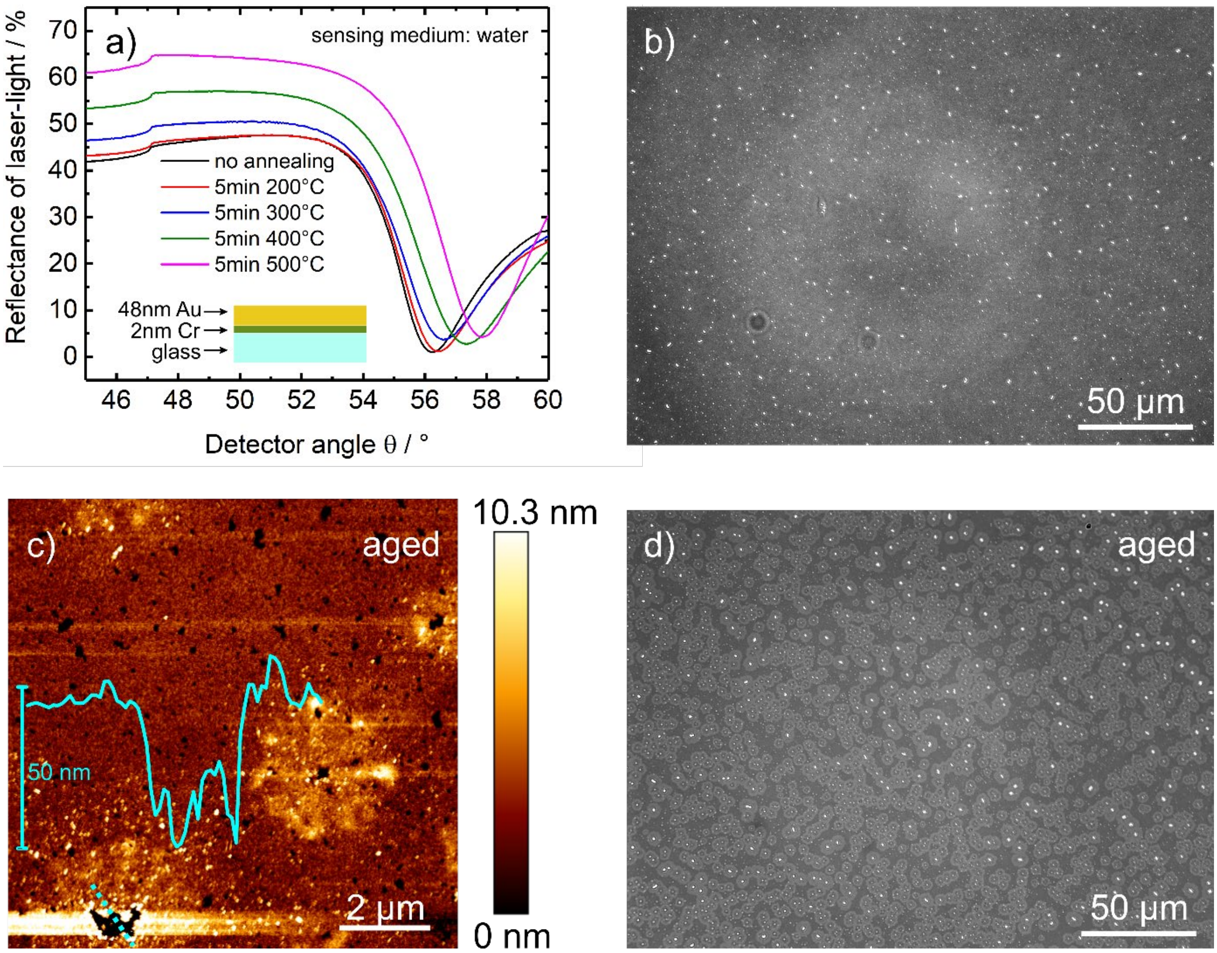

Figure S4. Simulated reflectance spectra in water showing the effect of varying layer thickness on the evolution of the spectral characteristics: (**a**) Effect of decrease in the thickness of the chromium adhesion layer. (**b,c**) Effect of increase in the thickness of chromium (**b**) and chromium oxide (**c**) layer accumulating on the gold surface. The insets present schemes of the composition of the multilayer used for the simulations.

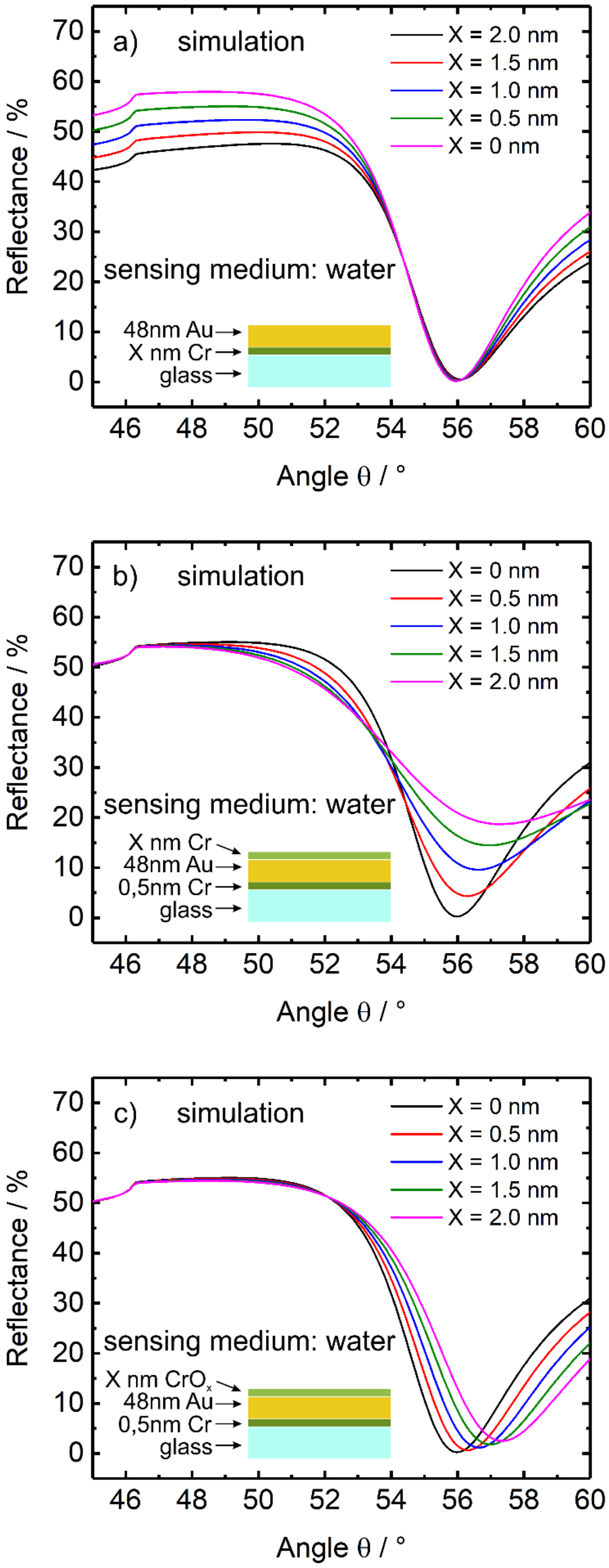

3. Development of optimized preparation procedure (MACE)

We have experimented several methods before arriving at the optimized MACE procedure. In a first attempt, we used the etchant (cerium ammonium nitrate solution) to remove the surface-migrated chromium (oxide). SPR-Au-chips that were annealed at 500 °C (in N_2) and etched using this etchant showed a good SPS curve even when reheated again to 500 °C. Such chips should then be suitable for the transfer of graphene. However, the holes present after a direct annealing of the Au chips at 500 °C still pose some challenges for obtaining an efficient graphene transfer. First, the increased surface roughness introduces stress in graphene, which may compromise the intactness of the graphene sheet when subjected to chemical or heat treatments. Secondly, the micro-holes in the gold-layer could cause faster degradation of the SPR-Au-chip as oxygen or electrolytes can now reach the underlayers directly. Finally, Au/Cr layers with holes had very limited lifetime as examined by the scotch tape test, which easily peeled away the defective metallic layer. [18] By contrast, as-prepared Au/Cr films were very robust to the scotch tape peeling test. These attempts led us to develop the 3-step MACE procedure.

Figure S5. Optical images in reflection mode (**a,c**) and transmission mode (**b,d**) of a MACE-treated SPR-Au/Gr chip showing the same graphene peripheral region before (**a,b**) and after (**c,d**) annealing to 500°C for 2 minutes under nitrogen atmosphere. Compare images in figure S1 where the samples did not undergo MACE-treatment.

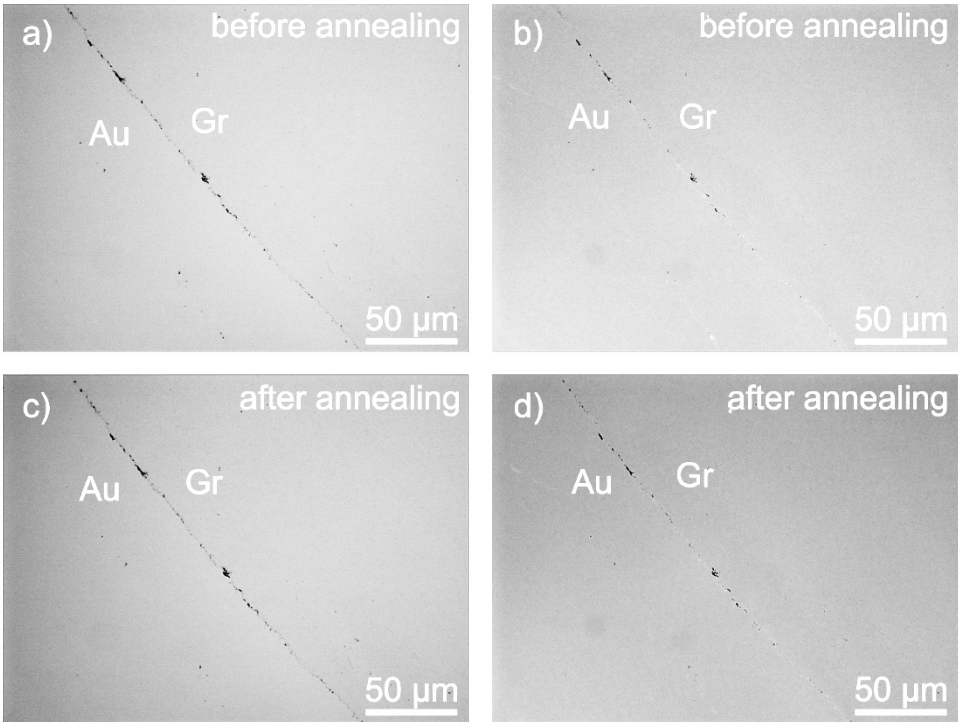

Figure S6. Electrochemical etching of migrated Cr / CrOx: (**a**) cyclic voltamograms recorded in 0.1 M H_2SO_4 under constant flow of electrolyte showing the electrochemical etching of chromium(oxide) for an SPR-Au-chip annealed at 500 °C (5 minutes under nitrogen atmosphere) connected as the working electrode. (**b**) sensorgram starting at 50% reflectance on the lower angle side of the SPS-curve of the same SPR-Au-chip showing the corresponding change in reflectance during the aforementioned potential cycling. (**c**) Reflectance spectra in water for an SPR-Au-chip annealed at 500°C (5 minutes under nitrogen atmosphere) before (black curve) and after (red curve) electrochemical etching of the chromium(oxide) layer.

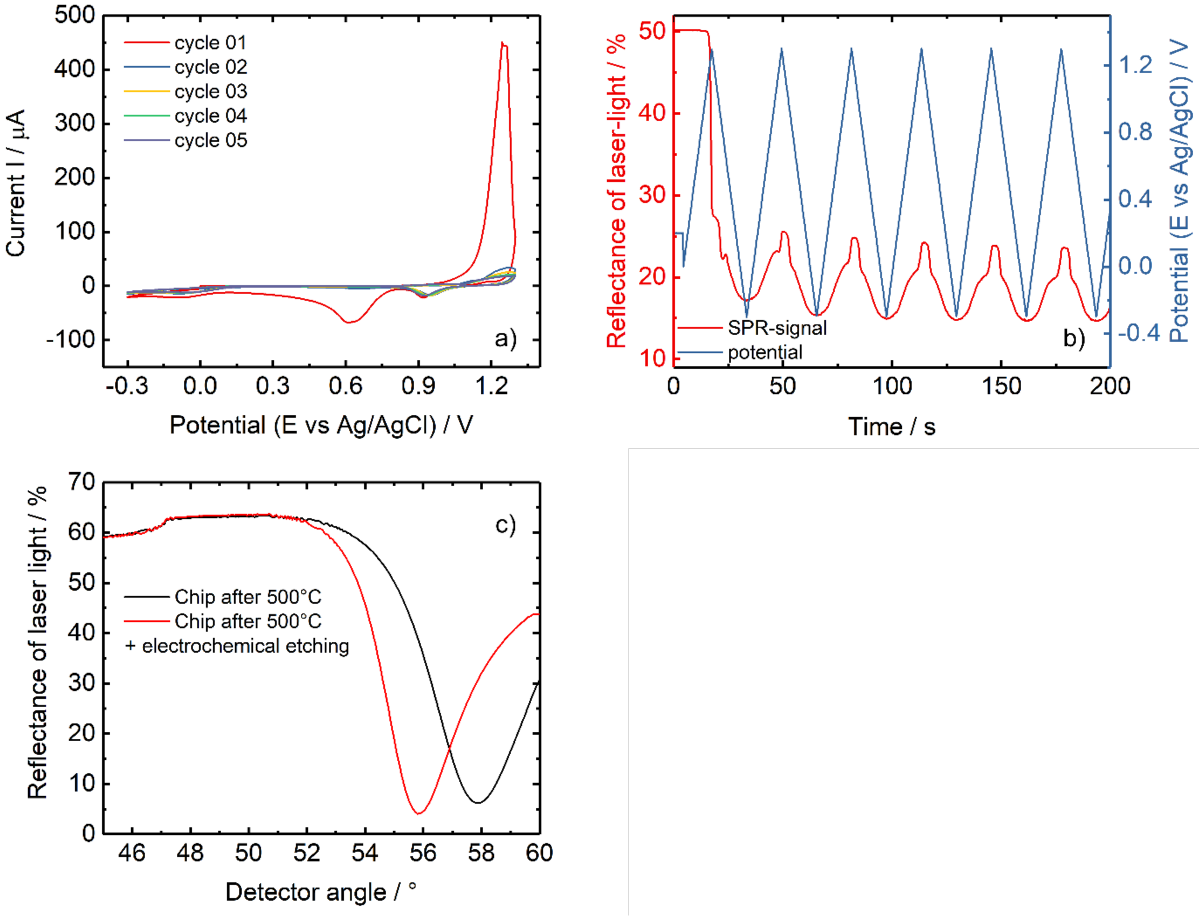

The SPS curves were unchanged for exposure to different electrolytes and cyclic voltammetry in a moderate range of -0.3 V to 0.5 V. After applying high oxidative potentials in a 0.1 M sulfuric acid electrolyte - as used often for cleaning of gold-electrodes - a strong anodic current at 1.1 V was detected (red curve in (a)), overlaid with the oxidation current of gold. Consecutive cycling towards 1.3 V while in sulfuric acid resulted in a steady position for the SPS curve minimum at an angle of around 56 ° and 5 % reflectance while maintaining the high maximum reflectance gained after the annealing step.

Figure S7. AFM images of the gold surface of SPR-Au-chips: (**a,d**) unheated SPR-Au-chips with a low initial roughness after deposition of the metal layers; (**b,e**) unheated SPR-Au-chips with a higher initial roughness after deposition of the metal layers; (**c,f**) SPR-Au-chips as in (**b,e**) but directly annealed at 500 °C (5 minutes under nitrogen atmosphere) and Cr-etched.

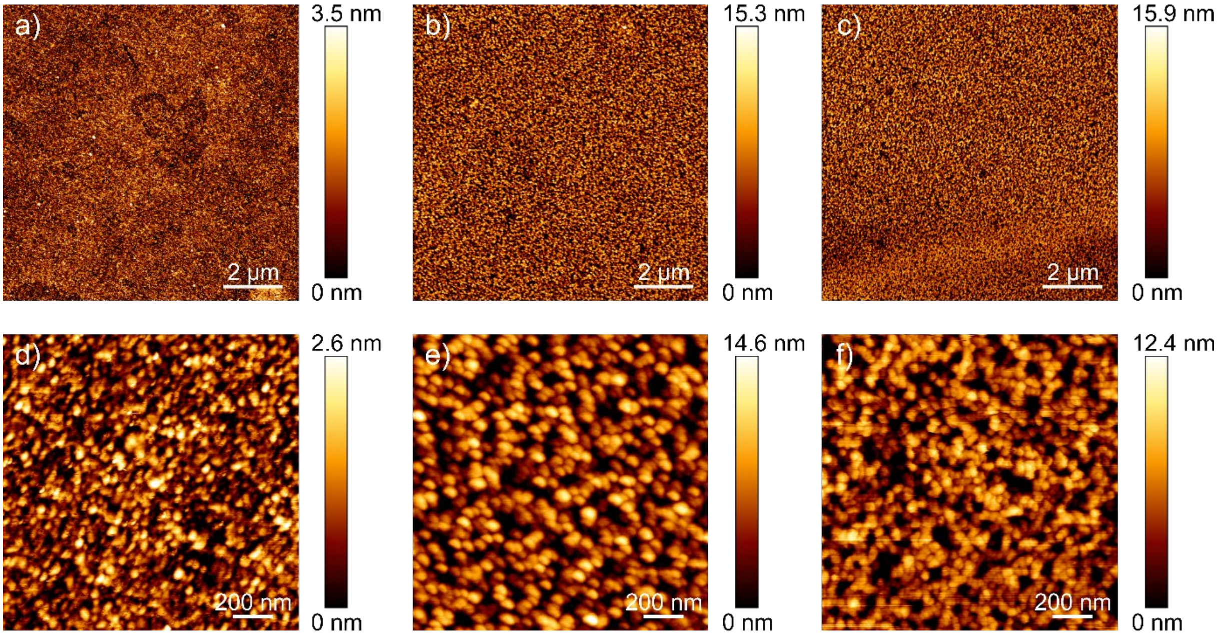

4. Spectroscopic evidence for the occurrence and removal of surface chromium (oxide) species

Evidence for the migration and oxidation of chromium in the Au/Cr films and the removal by etching is obtained by XPS and ToF-SIMS measurements. XPS spectra of an SPR-Au-chip heated to 500 °C were compared with the spectra of an SPR-Au-chip that was not undergoing any heat treatment and an SPR-Au-chip that was treated with the MACE procedure. Figure S8a shows the survey spectra of the three SPR-Au-chips. All spectra are dominated by peaks from elemental gold (the quantification results are shown in table S1). Prominent peaks related to chromium-species and oxygen are only present in the spectra of the SPR-Au-chip heated to 500°C (red curve). High resolution spectra obtained on this heat-treated chip (figure S8b) shows clearly the presence of different chromium oxide and hydroxide species (fit parameters are shown in table S2). On the other hand, the as-prepared SPR-Au-chip as well as the MACE chip show no traces of chromium (within the detection limit) as evident also from the high resolution spectra (figure S8c) normalized to the Au-$4f_{7/2}$ peak. This is a confirmation for the proposed model of chromium diffusing towards the surface during the heat treatment procedure. Furthermore, the effectiveness of the Cr-etching is also attested by these data, as there are no peaks of chromium species detectable after treatment of the chip using the MACE procedure.

The occurrence of migrated Cr(Ox) upon annealing and its removal after Cr(Ox)-etching were alos verified using ToF-SIMS. ToF-SIMS was chosen due to its extremely high surface sensitivity with a depth resolution of approximately 1 nm and its high mass resolution of $m/\Delta m$ = 11000. ToF-SIMS spectra of an SPR-Au-chip previously heated to 500 °C were recorded and compared with the spectra of an SPR-Au-chip heated to 500 °C and subsequently treated with Cr-etching solution. Figure S9 shows a summary of selected Au and Cr related ion peaks. From figures S9b, S9d, S9e and S9f it is quite clear that after Cr-etching, the amount of surface chromium has drastically reduced. The relatively smaller peak height for positive gold ions (figure S9a) is because the Bi^+-ion beam produces mainly negative gold ions (S9c) after impact on the gold-surface, while interaction of the beam with chromium leads predominantly to positive chromium ions (figure S9b). Strong signals originating from chromium-oxide species were mostly found in the negative ion scan (figures S9d to S9f). Comparison of the peak heights or areas can only give information of the change of content of one species before and after the treatments. The areas and heights cannot be translated to an actual composition of gold and chromium oxide in the surface domain due to the different formation probabilities of the detected species.

Figure S8. (**a**) XPS survey spectra of the surface of an as-prepared SPR-Au-chip (black curve), an SPR-Au-chip after annealing at 500 °C for 5 minutes under N_2 (red curve) and an SPR-Au-chip treated with the MACE procedure (blue curve). (**b**) High-resolution XPS spectra of the chromium-2p region along with the fits for chromium and its oxide-species for the heat-treated SPR-Au-Chip – red cruve in (**a**). (**c**) High-resolution XPS spectra of Cr-2p and Au-4f region of the three chips with the intensities normalized to the intensity of the Au-$4f_{7/2}$ peak.

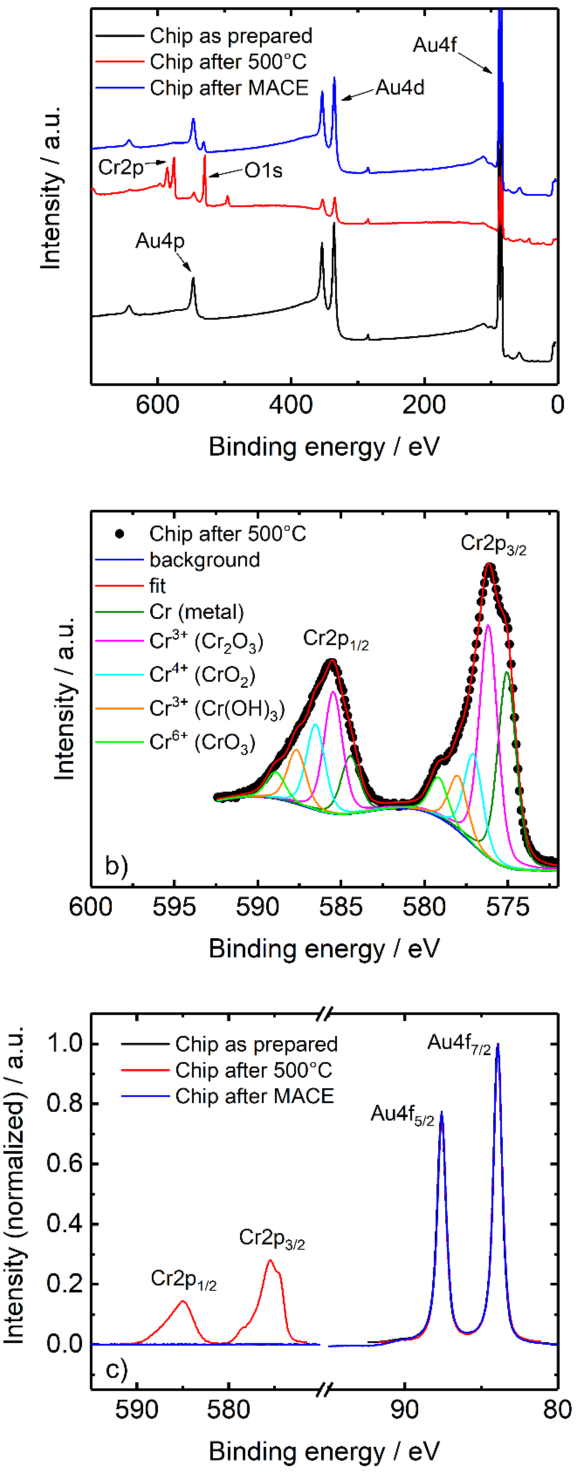

Table S1: Quantification of the elemental composition of the samples in at.%. The relative uncertainty is around 20% with a confidence interval of 95%.

**Sample**	**Au 4f**	**C 1s**	**O 1s**	**Cr 2p**	**Na 1s**
**As prepared**	84.2	13.8	2.0	n.d	n.d.
**After 500°C**	16.6	12.7	41.5	20.2	9.1
**After MACE**	85.2	12.3	2.4	n.d.	n.d.

n.d.: not detectable. The detection limit of Cr in a Au matrix is 1 at%.

Table S2: Fit parameters for Cr 2p peaks. The uncertainty in the binding energy is ± 0.2 eV.

Doublet name Cr2p	Peak height/ cps	Lorentzian	Position/ eV	FWHM/ eV	abs. Area/ cps·eV	rel. Area/ %
Cr(metal)	9916.1	0.3	575.0562	1.3	15197	20.22
	2937.7	0.3	584.4487	1.3	4569	6.08
Cr2O3	11772.6	0.3	576.1659	1.3	18165	24.17
	6168.8	0.3	585.4725	1.3	9585	12.75
CrO2	4597.1	0.3	577.0707	1.3	7116	9.47
	4336.1	0.3	586.5296	1.3	6726	8.95
Cr(OH)3	2785.1	0.3	577.9855	1.3	4321	5.75
	2748	0.3	587.668	1.3	4251	5.66
CrO3	2020.6	0.3	579.1492	1.3	3140	4.18
	1354.3	0.3	588.9141	1.3	2083	2.77

Figure S9. (**a,b**) Positive-ion-ToF-SIMS spectra (field of view = 100 µm x 100 µm, primary ion = Bi^+ 25 keV) showing sections for gold (**a**) and chromium (**b**) ions. (**c-f**) Negative-ion-ToF-SIMS spectra (field of view = 100 µm x 100 µm, primary ion = Bi^+ 25 keV) showing sections for gold ions (**c**) and ions of chromium-oxide species (**d-f**). Spectra were recorded as follows: red curve / (1): Cr/Au region subjected to annealing at 500 °C for 5 minutes under nitrogen atmosphere; blue curve / (2): Cr/Au region subjected to annealing as (1) and subsequent etching.

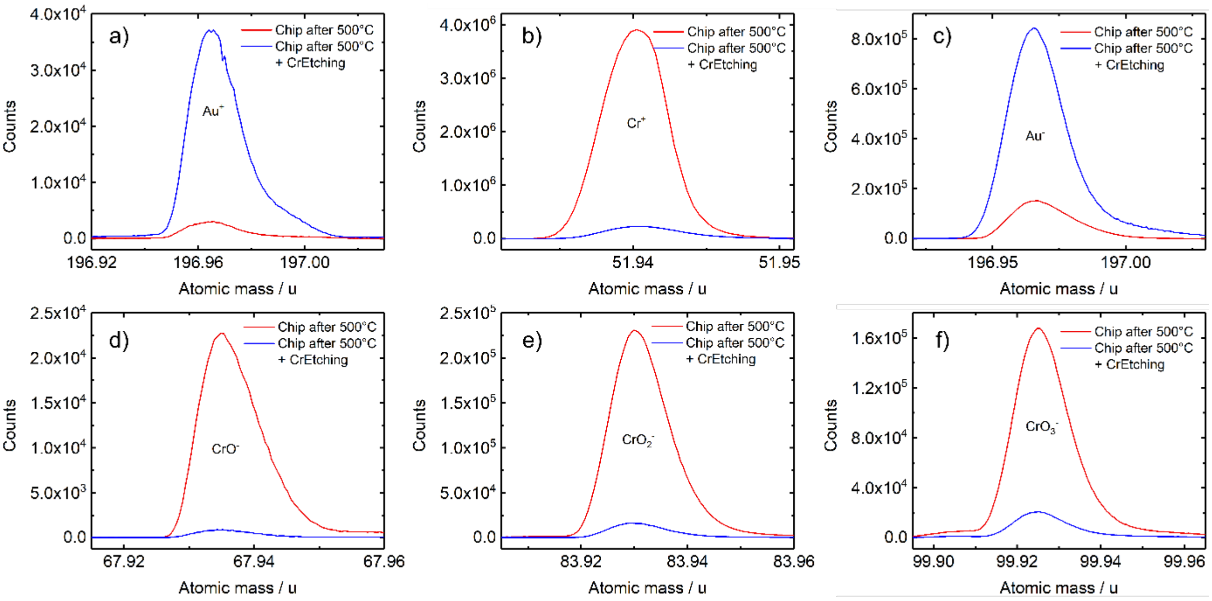

Table S3. The mass fractions of water and DMSO in water-DMSO mixtures and the calculated refractive indices of the various solutions. The numbers in the last column refer to the inset numbers in figure 3c.

H2O [g]	DMSO [g]	mass % H2O	mass % DMSO	refractive index	index number in figure
49.9876	0.0143	99.9714	0.0286	1.3317	1
49.9752	0.0251	99.9498	0.0502	1.3318	2
49.9499	0.0504	99.8992	0.1008	1.3318	3
49.8747	0.1251	99.7498	0.2502	1.3321	4
49.7495	0.2497	99.5006	0.4994	1.3324	5
49.5012	0.5004	98.9992	1.0008	1.3331	6
49.0005	0.9997	98.0006	1.9994	1.3346	7
48.0035	2.0004	95.9995	4.0005	1.3374	8
47.0003	3.0001	93.9998	6.0002	1.3403	9
46.0003	3.9998	92.0004	7.9996	1.3432	10
45.0000	4.9997	90.0005	9.9995	1.3460	11
44.0002	5.9996	88.0008	11.9992	1.3489	12

Figure S10. Resonance angle as a function of the refractive index of the sensing medium for an SPR-Au-chip (blue curve), an SPR-Au/Gr-chip (green curve), an SPR-Au-chip annealed via MACE (red curve) and an SPR-Au(MACE)/Gr-chip prepared with MACE-treatement where graphene was transferred and annealed at 500 °C for 2 minutes under nitrogen atmosphere (yellow curve).

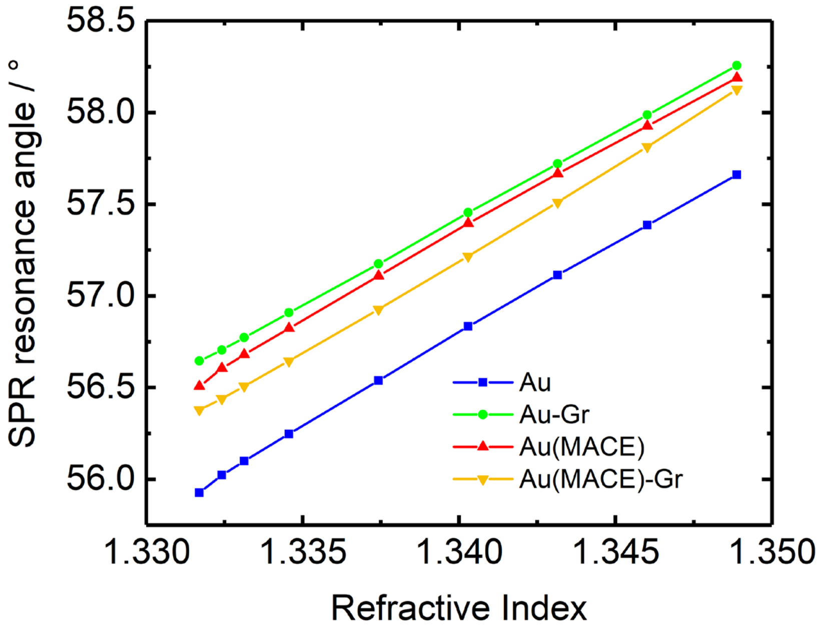

Table S4: Representative summary of different quality parameters extracted from SPR measurements on the indicated samples. The sample-to-sample variation in the values of extracted parameters was less than 5%. See experimental section for a definition of the various parameters.

Parameter	SPR-Au-chip	MACE-treated SPR-Au-chip	SPR-Au/Gr-chip (as prepared)	MACE-SPR-Au/Gr-chip after annealing
Dip strength [%]	48.3	65.4	45.7	65.2
Sensitivity [°/RIU]	97.2	100.1	97.2	103.0
QF [1/RIU]	61.1	65.2	59.7	60.8
DA [1/°]	0.63	0.65	0.61	0.59

Figure S11. SPS curves measured on an SPR-Au/Gr chip at the initial stage and after annealing the chip ten times at 500 °C in N_2 for five minutes each.

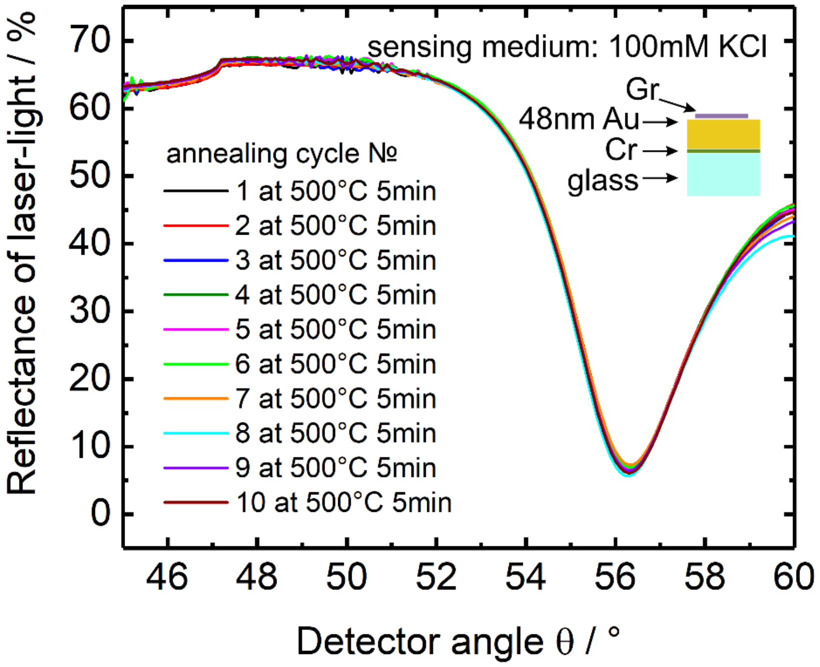

To test the stability of the MACE treated SPR-Au/Gr chips, a chip was annealed 10 times at 500 °C in nitrogen atmosphere for 5 minutes each. After each annealing cycle the quality of the chip was tested by (i) recording an SPR spectrum and (ii) by applying scotch tape on one corner and subsequently stripping it off to see if gold is peeled off from the glass substrate (scotch tape test). As can be seen in figure S11, the SPSspectra only show minor changes after each annealing step. The minor spectrum-to-spectrum variation might be a result of slight deviation in the SPR-setup after mounting the chip. The scotch tape test was negative after each annealing cycle showing that the adhesion of the gold onto the glass is not compromissed by the heat treatment even after 10 cycles.

Figure S12. SPR sensorgrams measured for the interaction of spiked avidin in 0.1% foetal bovine serum (FBS) at an SPR-Au/Gr-chip modified with BSA-biotin. The surface functionalization procedure is the same as shown in figure 4. The green shaded region shows the association phase, while the time span after that shows the dissociation phase. Two cycles of interaction were measured and the chip was regenerated in between by annealing to 500 °C in N_2. The repeatability of the kinetic response is clear, which show nearly the same behavior for both the cycles.

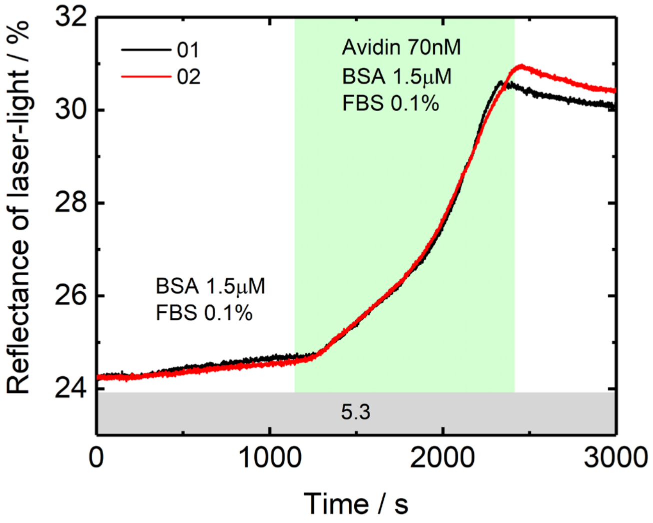

Figure S13. (a) SPR sensorgrams measured at an SPR-Au/Gr-chip prepared using the MACE procedure: binding of BSA to graphene in buffer. The same binding trial was repeated 6 times. After every cycle the chip was annealed to 500 °C in N_2. After cycle 3 and cycle 6 additionally avidin binding was monitored in a serum sample as shown in figure S12. After these two cycles the chip was annealed for 10 min. while after the other cycles the chip was annealed for 5 min. (b) Angular reflectance spectra in 10 mM phosphate buffer (pH 5.3) of the same SPR-chip before (cycle 0) and after the six cycles of sensing trials. After each of the annealing steps, the original spectrum is nearly completely restored. A sizeable deviation in the angular response is seen mainly after cycle 6.

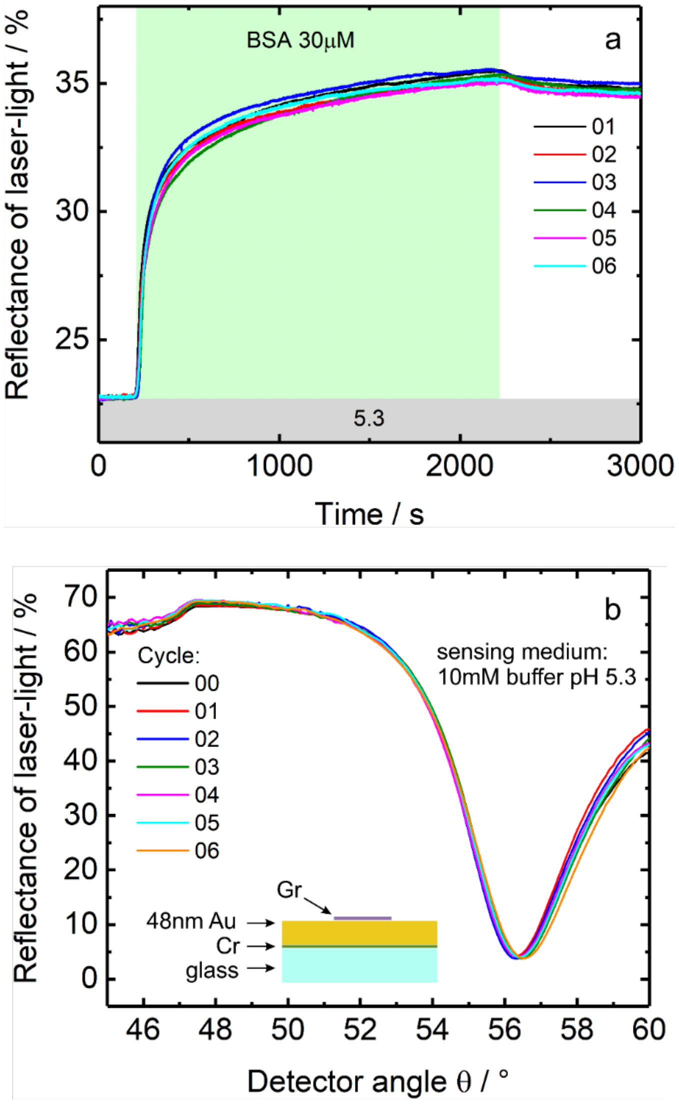

ESM References

1. Smith T. The hydrophilic nature of a clean gold surface. J Colloid Interface Sci. 1980;75:51-5.

2. Ekgasit S, Thammacharoen C, Knoll W. Surface plasmon resonance spectroscopy based on evanescent field treatment. Anal Chem. 2004;76:561-8.

3. Johnson P, Christy R. Optical constants of transition metals: Ti, V, Cr, Mn, Fe, Co, Ni, and Pd. Phys Rev B. 1974;9:5056-70.

4. Yakubovsky DI, Arsenin AV, Stebunov YV, Fedyanin DY, Volkov VS. Optical constants and structural properties of thin gold films. Opt Express. 2017;25:25574-87.
5. Al-Kuhaili MF, Durrani SMA. Optical properties of chromium oxide thin films deposited by electron-beam evaporation. Opt Mater. 2007;29:709-13.
6. Gesheva KA, Ivanova T, Szekeres AM, Trofimov O. Surface Characterization Of Chromium Oxide Thin Films in Dependence on CVD Growth Process Parameters. ECS Trans. 2007;2:229-36.
7. Mejard R, Thierry B. Systematic study of the surface plasmon resonance signals generated by cells for sensors with different characteristic lengths. PLoS One. 2014;9:e107978.
8. Rahman MS, Anower MS, Hasan MR, Hossain MB, Haque MI. Design and numerical analysis of highly sensitive Au-MoS2-graphene based hybrid surface plasmon resonance biosensor. Opt Commun. 2017;396:36-43.
9. Treebupachatsakul T, Boosamalee A, Chaithatwanitch K, Pechprasarn S. Generalized figure of merit for plasmonic dip measurement-based surface plasmon resonance sensors. Biomedical Optics Express. 2022;13:1784-800.
10. Liu F, Wang M, Chen Y, Gao J. Thermal stability of graphene in inert atmosphere at high temperature. J Solid State Chem. 2019;276:100-3.
11. Nan HY, Ni ZH, Wang J, Zafar Z, Shi ZX, Wang YY. The thermal stability of graphene in air investigated by Raman spectroscopy. J Raman Spectrosc. 2013;44:1018-21.
12. Huang Y, Qiu H, Wang F, Pan L, Tian Y, Wu P. Effect of annealing on the characteristics of Au/Cr bilayer films grown on glass. Vacuum. 2003;71:523-8.
13. Holloway PH. Gold/chromium metallizations for electronic devices. Gold Bull. 1979;12:99-106.
14. Thurner G, Holloway PH. Oxidation of Polycrystalline Chromium between 30°C and 400°C. Acta Phys Pol, A. 1992;81:273-83.
15. Palacio C, Mathieu HJ, Landolt D. AES, XPS and EELS study of the initial oxidation of polycrystalline chromium. Surf Sci. 1987;182:41-55.
16. Zhang H, Jin Y-X, Wang H, Kong F-Y, Huang H-P, Cui Y. Effects of annealing time on the structure, morphology, and stress of gold–chromium bilayer film. Chinese Physics B. 2016;25:104205.
17. Kenrick PS. Grain Boundary Diffusion Effects in Films of Gold on Chromium. Nature. 1968;217:1249-51.
18. Ghorbanpour M, Falamaki C. A novel method for the production of highly adherent Au layers on glass substrates used in surface plasmon resonance analysis: substitution of Cr or Ti intermediate layers with Ag layer followed by an optimal annealing treatment. Journal of Nanostructure in Chemistry. 2013;3:1-7.
19. Mariotti MP, Riccardi CdS, Fertonani FL, Yamanaka H. Strategies for developing NADH detector based on meldola blue in different immobilization methods: a comparative study. J Braz Chem Soc. 2006;17:689-96.
20. Titoiu AM, Lapauw M, Necula-Petrareanu G, Purcarea C, Fanjul-Bolado P, Marty J-L, et al. Carbon Nanofiber and Meldola Blue Based Electrochemical Sensor for NADH: Application to the Detection of Benzaldehyde. Electroanal. 2018;30:2676-88.
21. Kaya NS, Yadav A, Wehrhold M, Zuccaro L, Balasubramanian K. Binding Kinetics of Methylene Blue on Monolayer Graphene Investigated by Multiparameter Surface Plasmon Resonance. ACS Omega. 2018;3:7133-40.
